# The Potential of Using Immobilized Xylanases to Enhance the Hydrolysis of Soluble, Biomass Derived Xylooligomers

**DOI:** 10.3390/ma11102005

**Published:** 2018-10-17

**Authors:** Jinguang Hu, Joshua Davies, Yiu Ki Mok, Claudio Arato, John N. Saddler

**Affiliations:** 1 Forest Products Biotechnology/Bioenergy Group, Department of Wood Science, Faculty of Forestry, The University of British Columbia, 2424 Main Mall, Vancouver, BC V6T 1Z4, Canada; jinguang.hu@ucalgary.ca (J.H.); tonyykmok@gmail.com (Y.K.M.); 2 Department of Chemical and Petroleum Engineering, University of Calgary, 2500 University Dr. NW, Calgary, AB T2N 1N4, Canada; 3 S2G BioChemicals, 4250 Wesbrook Mall, Vancouver, BC V6T 1W5, Canada; jdavies@s2gbiochem.com (J.D.); carato@Fortressab.com (C.A.)

**Keywords:** biorefining, xylanases, enzyme immobilization, biomass pre-treatment, xylooligomers

## Abstract

Earlier work had indicated that enzyme-mediated hydrolysis of xylooligomer-rich water-soluble streams (derived from steam pre-treated wheat straw) resulted in the effective production of xylose which was subsequently used to produce bio-glycol. In the work reported here, both the thermostability and recyclability of xylanases were significantly improved by covalent immobilizing the enzymes onto alginate beads. The immobilized xylanases showed a lower hydrolytic potential (~55% xylooligomer conversion) compared to the commercial xylanase cocktail HTec3 (~90% xylooligomer conversion) when used at the same protein loading concentration. This was likely due to the less efficient immobilization of key higher molecular weight enzymes (>75 kDa), such as β-xylosidases. However, enzyme immobilization could be improved by lowering the glutaraldehyde loading used to activate the alginate beads, resulting in improved hydrolysis efficacy (~65% xylooligomer conversion). Enzyme immobilization improved enzyme thermostability (endoxylanase and β-xylosidase activities were improved by 80% and 40%, respectively, after 24 h hydrolysis) and this allowed the immobilized enzymes to be reused/recycled for multiple rounds of hydrolysis (up to five times) without any significant reduction in their hydrolytic potential.

## 1. Introduction

One of the key challenges in establishing an effective “biorefinery” is to biochemically deconstruct the polysaccharides within the lignocellulosic substrate to the “sugar platform” which can subsequently be used to produce a range of chemicals and fuels [1,2]. Due to the recalcitrant nature of biomass, a physicochemical pre-treatment process is typically required to open up the tightly packed cell wall structure to facilitate the access of the enzymes to the target polysaccharides, cellulose and hemicellulose. In many of the industrial relevant pre-treatment processes (e.g., steam explosion, hydrothermal pre-treatment, diluted acid pre-treatment, etc.) that have been used, increased cellulose accessibility has been achieved by solubilizing a large portion of hemicellulose [3,4]. However, most of this solubilized hemicellulose remains in an oligomeric form, which somewhat limits their further valorization [5,6]. However, in earlier work, hemicellulose enzymes were successfully used to hydrolyze these hemicellulose-derived oligomers to monomeric sugars, particularly when compared with traditional acid hydrolysis processes [7].

Although successful, this previous work had indicated that some of the enzymes were precipitated/denatured and that agitation was not required for the optimized enzymatic hydrolysis process [7]. Thus, it is possible that an enzyme immobilization strategy could be used to improve both the thermostability of the enzymes and our ability to recycle/reuse the enzymes, resulting in reduced enzyme-related costs. There are several ways to immobilize xylanase enzymes, including physical adsorption, covalent binding, ionic binding and entrapment of enzymes on solid materials (Table 1) [8,9,10,11,12]. To briefly summarize, physical adsorption is characterized by enzyme adsorption to the solid substrate via hydrophobic interactions, hydrogen bonding, van der Waals forces, or electrostatic interactions [11]. Covalent binding involves the linkage of appropriate reactive groups of the enzyme structure with a support while ensuring the active site of the enzyme in unobstructed [8,9]. Entrapment is typically characterized by the encapsulation of the enzymes within an inorganic or polymeric matrix, where the free flow of substrate or product molecules from the bulk medium to the enzymes is not restricted [10,11]. Ionic immobilization involves the cation or anion exchanger for binding enzymes to solid supports [12].

Of these various methods, the covalent binding of xylanases onto alginate beads appeared to be the most promising, since it offered a simple method of enzyme immobilization and resulted in the highest immobilization yield as compared to other methods (Table 1). Alginate beads have been routinely used for enzyme entrapment and previous work has shown that the covalent immobilization of xylanases on alginate beads improved enzyme thermostability [9]. It was also apparent that the immobilized xylanase enzyme activity remained high, even after multiple rounds of enzyme recycling (Table 1). Most previous studies have investigated the immobilization of one purified xylanase enzyme applied in a relatively “simple” hydrolysis environment [8,9,10,11,12]. In contrast, an enzyme cocktail composed of multiple xylanase enzymes (at least containing endo-xylanases and β-xylosidases) with different physical and functional properties is more likely to be used in more complex environments that would contain various soluble components derived from biomass pre-treatment.

In the work reported below, the surface covalent immobilization of a commercial xylanase preparation (HTec3) on alginate beads and the hydrolytic potential of these immobilized xylanases towards the xylooligomers present in pre-treatment-derived liquors were assessed. The enzyme immobilization efficacy was optimized by adjusting the glutaraldehyde loading. The stability and recyclability of immobilized xylanases were also assessed. This approach showed considerable potential to reduce the enzyme dosages/cost required to achieve effective xyloosaccharide hydrolysis.

## 2. Materials and Methods

### 2.1. Substrate Pre-treatment and Water Soluble Component/Oligosaccharide Production

The water-soluble fractions, obtained after hydrothermally pre-treated autohydrolysis of wheat straw, was provided courtesy of Beta-Renewables (Tortona, AL, Italy). Steam pre-treatment conditions had been optimized to maximize both hemicellulose recovery and ease of enzymatic hydrolysis of the cellulose-rich, water-insoluble fraction [4,13]. The water soluble fraction contained the monomeric sugars arabinose (1.0 g/L), galactose (0.3 g/L), glucose (0.3 g/L), xylose (1.5 g/L), and xylooligomeric sugars (23.6 g/L). It also contained acid soluble lignin and acid insoluble lignin at 4.5 g/L and 3.7 g/L, respectively.

### 2.2. Sodium Alginate Beads Production and Activation

The sodium alginate beads were produced by dropping a 2% (*w*/*v*) sodium alginate solution through a syringe connected with an 18-gauge needle into 0.02 M CaCl_2_ solution [9]. The ratio between the alginate and the CaCl_2_ solution was 1:2 (*v*/*v*). The beads were subsequently stored at 4 °C overnight to allow them to harden.

The alginate beads were further activated by dipping them into a 0.5–12% *w*/*v* glutaraldehyde solution. The activation process was carried out at room temperature under orbital stirring (150 rpm) in a benchtop hybridization incubator (Combi-H12 hybridization incubator, FINEPCR, Seoul, Korea) for 3 h using a ratio of 1:10 beads:glutaraldehyde solution [9,10,11]. After activation, the beads were thoroughly washed with distilled water to remove any unbound glutaraldehyde. The beads were immerged in 1 L distilled water each time and mixed for 1 h at room temperature using a magnetic stir bar. The water was subsequently purged and the beads were collected in a metal mesh and immerged in fresh water. This was repeated five times to ensure all of the unbounded glutaraldehyde was washed out.

### 2.3. Enzyme Immobilization

The commercial xylanase enzyme preparation HTec3 (Novozymes, Franklington, NC, USA) was diluted in 50 mM sodium acetate buffer and mixed with the activated beads at a ratio of 1:1 (*v*/*w*) [9,10,11,12]. The enzyme and bead mixture was then mixed at 200 rpm for 2 h using an orbital shaker. After activation, the beads were thoroughly washed with distilled water to remove any unbound enzymes. The immobilization efficiency was determined by quantifying the protein concentration in solution after immobilization using the ninhydrin method [14]. The immobilization yield was calculated based on enzyme mass specific yield, which has been defined as the mass of protein bound to the carrier according to Liese and Hilterhaus [15]. The ninhydrin assay was selected due to its specificity for protein and its low level of interference from compounds present in lignocellulosic substrates [7]. Sodium Dodecyl Sulfate Polyacrylamide Gel Electrophoresis (SDS-PAGE) gels were also performed and stained with Coomassie Blue G250 dye to determine the composition of the enzymes that were immobilized.

### 2.4. Enzyme Hydrolysis and Recycle

The sugar composition of xylooligomer-enriched water-soluble fractions derived from industrial relevant biomass pre-treatment processes was determined using high performance anion exchange chromatography (Dionex DX-3000, Sunnyvale, CA, USA) as described previously [13]. The hydrolysis of xylooligomer-enriched water-soluble fractions with immobilized enzymes was carried out at a loading of ~2.5 mg proteins per gram of oligomeric xylose at 50 °C, pH 4.3 in a bench top hybridization incubator (Combi-H12 hybridization incubator, FINEPCR, Seoul, Korea) without shaking according to our previously optimized hydrolysis condition [8]. After enzymatic hydrolysis, the hydrolysate was separated from the alginate beads by filtration through a 20-micron metal mesh and was heated at 100 °C for 10 min to inactivate the possibly released enzymes. The supernatants were collected after centrifugation at 13,000 rpm (16,000 g) for 10 min and stored at −20 °C. The concentrations of monomeric sugars were measured using High Performance Liquid Chromatography (HPLC) as described earlier [13]. The alginate beads with immobilized enzymes were collected and washed with distilled water for another cycle of hydrolysis in the enzyme recycling work. All of the hydrolysis reactions were run in triplicate and the mean value was reported.

Since one molecule of water was added to form one molecule of xylose during xylooligomer hydrolysis, the xylooligomer conversion yield was calculated assuming a xylose concentration of 0.88/(xylooligomer concentration) according to Formula (1) [7].

(1) $$\mathrm{Xylose}\;\mathrm{yield}\;\left(\%\right)=\frac{\mathrm{xylose}\;\left(g/L\right)\;\times \;0.88}{\mathrm{xylooligomer}\;\left(g/L\right)}\;\times \;100$$

### 2.5. Enzyme Activity Assay

The xylanase and β-xylosidase activities of the complete enzyme mixture and immobilized enzymes were also compared over the course of 24 h to determine any changes in their respective stability and hydrolytic performance. Briefly, β-xylosidase activities were determined by using p-nitrophenyl-β-D-xylobioside (p-NPX) as substrates according to [16,17]. For xylanase activities, birchwood glucoronoxylan was dissolved in 50 mM sodium acetate buffer (pH 4.3) by stirring overnight at room temperature, then 70 μL dissolved xylan substrates were added in microplates with 30 μL of the appropriately diluted enzyme samples and mixed in an incubator at 400 rpm (PHMP Thermoshaker for Microplates, Thomas Scientific Swedesboro, NJ, USA) for various incubation times at 50 °C. The enzymatic reaction was stopped by adding 200 μL of 3,5-dinitrosalicylic acid (DNS) reagent after exactly 5 min, 10 min, 20 min and 30 min incubation. Afterwards, the microplates were placed in an oven at 105 °C and boiled for 30 min, and the reducing sugar content of the samples were analyzed by measuring the absorbance at 540 nm. Xylose standards were used for calibration. The reducing sugar released (μmol) at different hydrolysis times was plotted, and the enzyme activity (μmol/min) was determined by the slope of the linear phase of the hyperbolic curve. The relative xylanase activity was calculated according to Formula (2).

(2) $$\mathrm{Relative}\;\mathrm{xylanase}\;\mathrm{activity}\;\left(\%\right)=\frac{\mathrm{xylanase}\;\mathrm{activity}\;\mathrm{after}\;\mathrm{hydrolysis}\;\left(\mathrm{IU}/\mathrm{mg}\right)}{\mathrm{orignial}\;\mathrm{xylanase}\;\mathrm{activity}\;\left(\mathrm{IU}/\mathrm{mg}\right)}\;\times \;100$$

## 3. Results and Discussion

### 3.1. Covalent Surface Immobilization of Xylanases on Alginate Beads for Xylooligomer Hydrolysis

**Figure 1:**
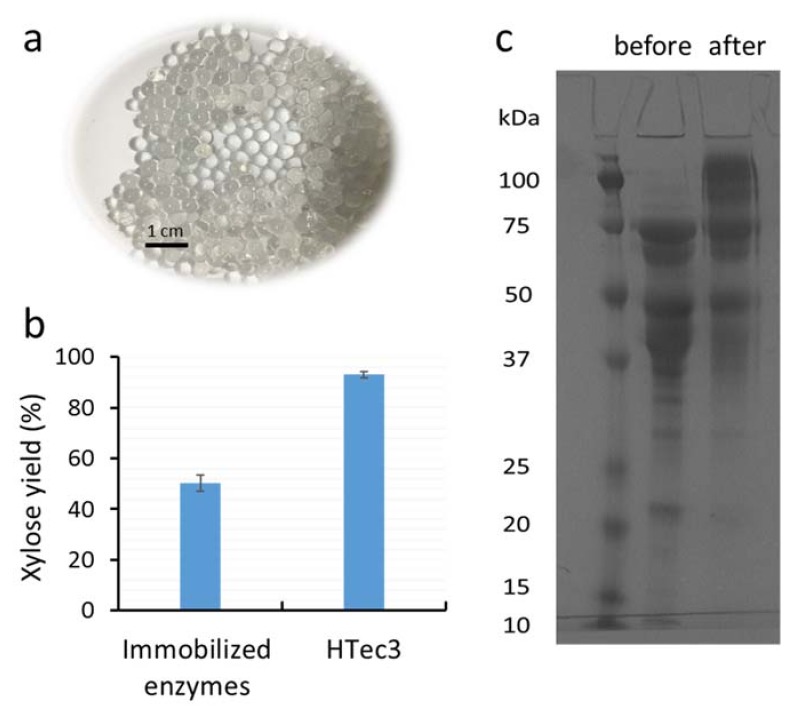
(**a**) Alginate beads activated with glutaraldehyde (3%, *w*/*w*) for enzyme immobilization; (**b**) the xylose yield of xylooligomer-enriched pre-treatment liquors after 24 h hydrolysis using either immobilized HTec3 (xylanases) or non-immobilized HTec3; and (**c**) SDS-PAGE of the HTec3 enzymes remaining in solution before (left column) and after immobilization (right column).

Alginate beads were produced and activated with glutaraldehyde (3%, *w*/*w*) to achieve the covalent surface immobilization of xylanase enzymes (Figure 1a). The yield when immobilizing the commercial xylanase preparation HTec3 was approximately 65% (mass specific yield), which was slightly lower than previous reports (Table 1) [8,9,10,11,12]. This lower immobilization yield was likely due to differences in the enzymes, as most previous studies only optimized immobilization conditions for one purified enzyme (Table 1). When the hydrolytic potential of the surface-immobilized enzymes was evaluated after addition to the xylooligomer-enriched water-soluble streams (~23 g xylooligomer per liter) [7], the hydrolysis yield of the xylooligomer was about 55% (Figure 1b). This hydrolysis yield was lower than what was previously reported using the original HTec3 at the same enzyme loading concentration (~90% xylooligomer conversion) [7].

To try to determine why the immobilized enzymes resulted in a lower hydrolysis yield than the original xylanase preparation HTec3, the composition of the immobilized xylanase enzymes was next assessed by SDS-PAGE analysis. After enzyme immobilization, the enzymes remaining in solution were assessed via SDS-PAGE. By comparing with the control (original enzyme solution before immobilization), we could then distinguish which types of xylanase enzymes were preferentially immobilized onto the alginate beads. It was apparent that the higher molecular weight enzymes (>75 kDa) in HTec3 did not bind efficiently to the activated alginate beads, as most of this protein band remained in the liquid phase after attempted immobilization (Figure 1c). β-xylosidases typically have a molecular weight between 80–120 kDa [18,19] and they play a key role in the hydrolysis of xylobiose or short xylooligomers to monomeric xylose. Thus, the reduced binding of β-xylosidases during immobilization was likely a major reason behind the reduced hydrolysis yield of immobilized enzymes. In addition, the yield and efficiency of the immobilized xylanase was likely further reduced by the slow inward and outward diffusion of the substrates and hydrolytic products, respectively. It was apparent that the catalytic performance of the xylanase was slightly decreased as a result of immobilization due to the increased Km value, although the change in Vmax was marginal. Thus, further optimization of the immobilization protocol will likely be required for complex enzyme mixtures such as HTec3.

### 3.2. Optimization of the Glutaraldehyde Loading Concentration for Enzyme Immobilization

**Figure 2:**
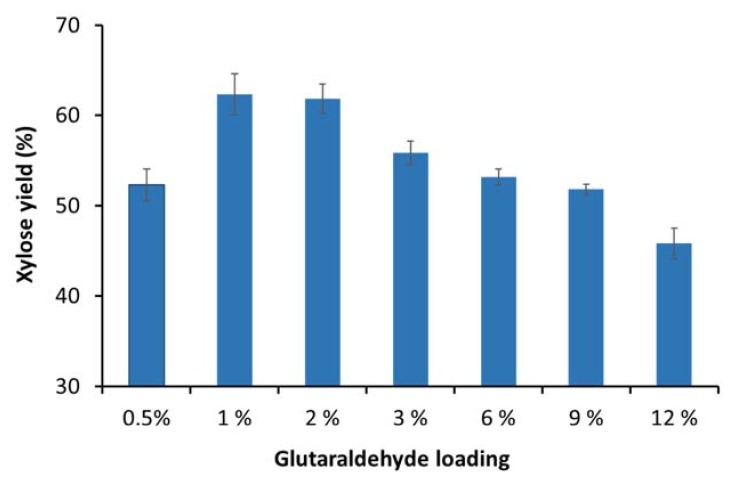
Hydrolysis of xylooligomer-enriched pre-treatment liquors after 24 h using immobilized xylanase enzymes on activated alginate beads with various glutaraldehyde concentrations.

Previous work has shown that enzyme immobilization efficacy can be significantly enhanced by optimizing the glutaraldehyde dose used for alginate bead activation [8,9,10]. Therefore, to try to improve the immobilization efficiency, various glutaraldehyde loading concentrations (0.5–12%) were assessed during the activation of the alginate beads. Enzyme immobilization was carried out after alginate bead activation and the immobilized enzymes were then used to hydrolyze the xylooligomer-enriched pre-treatment-derived liquid. It was apparent that the highest hydrolysis yields were achieved with the alginate beads that were activated with 1% glutaraldehyde, as about 65% of the xylooligomers could be hydrolyzed to xylose after 24 h hydrolysis (Figure 2). However, over the range of glutaraldehyde loading concentrations assessed, most of the β-xylosidases still did not bind to the alginate beads (data not shown). It was apparent that glutaraldehyde loading concentration could be reduced three-fold (from 3% to 1% glutaraldehyde) [7] with increased immobilization efficacy (Figure 2) as compared to the published protocol for immobilization of a purified xylanase from *Aspergillus niger* by Pal and Khanum (2011) [9]. The conditions that were found to be optimal for HTec3 immobilization on alginate beads were slightly different from previous studies. This was likely due to several factors, such as the HTec3 preparation containing multiple enzymes, the xylanases in HTec3 belonging to different glycoside hydrolase families and the non-enzyme components in HTec3 interfering with the surface immobilization process.

### 3.3. The Stability of Major Xylanases Before and After Immobilization

**Figure 3:**
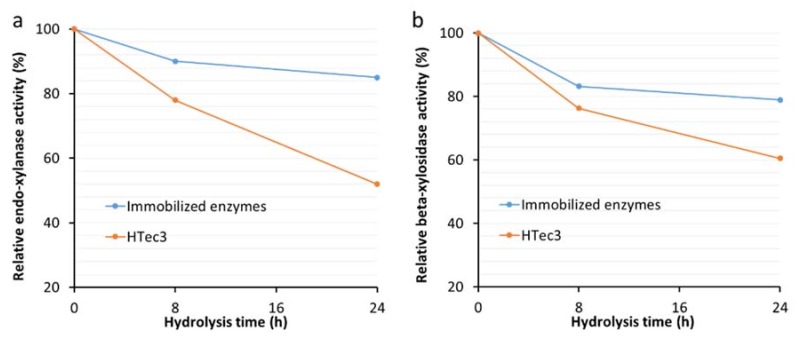
Relative endo-xylanase (**a**) and beta-xylosidase (**b**) activities of immobilized HTec3 xylanases and non-immobilized HTec3 xylanases over 24 h.

One of the advantages of enzyme immobilization is to improve enzyme thermostability during the time course of enzymatic hydrolysis [20,21]. Further analysis of the major enzymatic activities of immobilized xylanases (endo-xylanase and β-xylosidase activities) revealed that they retained approximately 90% and 85% of their endo-xylanase and beta-xylosidase activities, respectively, after 24 h incubation at enzymatic hydrolysis conditions (50 °C in 50 mM sodium acetate buffer) (Figure 3a,b). In contrast, the original commercial enzyme preparation HTec3 only retained about 50% and 60% of its endo-xylanase and beta-xylosidase activities, respectively, after 24 h incubation under the same conditions (Figure 3a,b).

It is generally acknowledged that the low thermostability of xylanases has been a major limitation in its application in traditional pulp and paper and evolving biorefinery industries [22,23,24]. Thus, the improved stability of xylanases via immobilization could increase the interest in various xylanase applications.

### 3.4. Assessing the Recyclability of Immobilized Xylanases over Multiple Rounds of Hydrolysis

**Figure 4:**
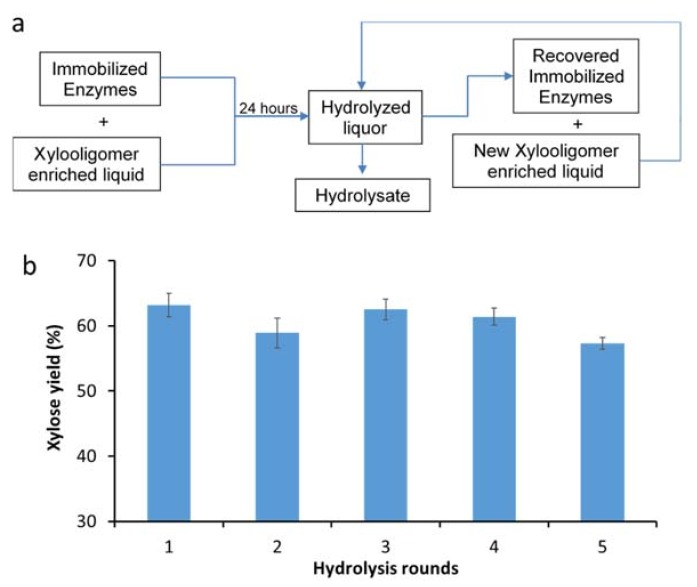
(**a**) Immobilized xylanase recycling protocol; (**b**) xylose yield from xylooligomer-enriched pre-treatment liquors over five rounds of recycling immobilized xylanases.

The immobilized xylanases were subsequently assessed for their recyclability over multiple rounds of hydrolysis. A simple enzyme recycling strategy was developed where the immobilized enzymes were used to hydrolyze xylooligomer-enriched pre-treatment-derived liquid over 24 h. This was followed by the removal of the hydrolyzed liquor (by filtration) and the addition of a fresh xylooligomer solution to the immobilized xylanases (Figure 4a). It was apparent that similar hydrolysis yields (~60%) were achieved, even after five rounds of immobilized enzyme recycling carried out over the course of five days (Figure 4b). Aside from the less effective immobilization of β-xylosidases, the lower degree of xylooligomer hydrolysis by using immobilized enzymes could also be due to the inhibitory compounds derived from both pre-treatment and enzymatic hydrolysis (i.e., soluble monomeric sugars, phenolics, furans, extractives, etc.) [25]. These inhibitory effects could likely be mitigated by various detoxification strategies, such as continuous product removal, chemical detoxification and activated carbon adsorption.

## 4. Conclusions

Commercial xylanase enzymes could be covalently immobilized onto alginate beads and their immobilization efficiency could be adjusted by varying the glutaraldehyde loading concentration used for bead activation. Although the immobilized xylanases had a slightly lower hydrolytic potential towards the xylooligomer-enriched water-soluble fraction (likely due to the less effective immobilization of the higher molecular weight β-xylosidases), they showed much higher thermostability and recyclability. This allowed them to be reused for multiple runs of hydrolysis without sacrificing the enzyme’s hydrolytic potential. The xylanase immobilization strategy could be further improved by using different supporting materials to provide a larger surface area and/or allow the inside covalent immobilization of xylanases.

## Figures and Tables

**Table 1:** Summary of the xylanase immobilization methods and their effect on enzyme recyclability [8,9,10,11,12].

Immobilization Method	Immobilization Support	Immobilization Yield (%)	Recyclability	Reference
Covalent binding	Polyaniline via glutaraldehyde	N/A	>72% of its original activity after 15 recycling rounds	[8]
	Glutaraldehyde activated alginate beads	>91	>85% of its original activity after five recycling rounds	[9]
	Polymethyl methacrylate (PMMA) nanofiber membrane (NFM) activated with glutaraldehyde	90	~80% of its original activity after 11 recycling rounds	[10]
	HP-20 (styrene-divynilbenzene adsorbent resin) with glutaraldehyde	42	~70% of its original activity after 11 recycling rounds	[11]
Ionic binding	Q-sepharose	45	~70% of its original activity after 11 recycling rounds	[11]
	Dowex-50W	24.5–47.4	18% of its original activity after 40 days	[12]
Physical adsorption	Chitin	35	<50% of its original activity after four recycling rounds	[11]
	Tannin-chitosan	37.7–69.3	33% of its original activity after 40 days	[12]
Entrapment	Gelatin	40	<50% of its original activity after four recycling rounds	[11]
